# Polydatin Attenuates Neuronal Loss *via* Reducing Neuroinflammation and Oxidative Stress in Rat MCAO Models

**DOI:** 10.3389/fphar.2019.00663

**Published:** 2019-06-26

**Authors:** Fawad Ali Shah, Lina Al Kury, Tao Li, Alam Zeb, Phil Ok Koh, Fang Liu, Qiang Zhou, Ishtiaq Hussain, Arif Ullah Khan, Yuhua Jiang, Shupeng Li

**Affiliations:** ^1^State Key Laboratory of Oncogenomics, School of Chemical Biology and Biotechnology, Shenzhen Graduate School, Peking University, Shenzhen, China; ^2^Riphah Institute of Pharmaceutical Sciences, Riphah International University, Islamabad, Pakistan; ^3^College of Natural and Health Sciences, Zayed University, Abu Dhabi, United Arab Emirates; ^4^Department of Forensic Medicine, School of Medicine, Xi’an Jiaotong University, Xi’an, China; ^5^Department of Anatomy, College of Veterinary Medicine, Research Institute of Life Science, Gyeongsang National University, Jinju, South Korea; ^6^Campbell Research Institute, Centre for Addiction and Mental Health, Toronto, ON, Canada; ^7^Department of Psychiatry, University of Toronto, Toronto, ON, Canada; ^8^Department of Pharmacy, AUST, Abbottabad, Pakistan; ^9^Cancer Centre, The Second Hospital of Shandong University, Jinan, China

**Keywords:** polydatin, ischemic stroke, neuroinflammation, oxidative stress, neuronal death

## Abstract

Ischemic stroke is characterized by permanent or transient obstruction of blood flow, which initiates a cascading pathological process, starting from acute ATP loss and ionic imbalance to subsequent membrane depolarization, glutamate excitotoxicity, and calcium overload. These initial events are followed by neuroinflammation and oxidative stress, eventually causing neuronal neurosis and apoptosis. Complicated interplays exist between these steps happening across various stages, which not only represent the complicated nature of ischemic pathology but also warrant a detailed delineation of the underlying molecular mechanisms to develop better therapeutic options. In the present study, we examined the neuroprotective effects of polydatin against ischemic brain injury using a rat model of permanent middle cerebral artery occlusion (MCAO). Our results demonstrated that polydatin treatment reduced the infarction volume and mitigated the neurobehavioral deficits, sequentially rescued neuronal apoptosis. Ischemic stroke induced an elevation of neuroinflammation and reactive oxygen species, which could be attenuated by polydatin *via* the reduced activation of p38 mitogen-activated protein kinase and c-Jun N-terminal kinase. In addition, polydatin upregulated the endogenous antioxidant nuclear factor erythroid 2-related factor 2, heme oxygenase-1, the thioredoxin pathway, and eventually reversed ischemic-stroke-induced elevation of ROS and inflammation in ischemic cortical tissue. The diverse and broad actions of polydatin suggested that it could be a multiple targeting neuroprotective agent in ameliorating the detrimental effects of MCAO, such as neuroinflammation, oxidative stress, and neuronal apoptosis. As repetitive clinical trials of neuroprotectants targeting a single step of stroke pathological process have failed previously, our results suggested that a neuroprotective strategy of acting at different stages may be more advantageous to intervene in the vicious cycles in MCAO.

## Introduction

Stroke is the second leading cause of mortality worldwide and ischemic stroke accounts for approximately 80% of stroke incidence. Resulting from an occlusion of a major cerebral artery [most often middle cerebral artery (MCA)] or its branches, ischemic stroke is characterized by permanent or transient obstruction of blood flow, impeding the delivery of oxygen and essential nutrients to the brain. Tissue plasminogen activator (tPA) is the only FDA-approved drug to reverse the adverse effects of stroke by recanalizing obstructed vessels (i.e., the vascular strategy for combating stroke). However, many clinical limitations are associated with tPA administration, strengthening the need for alternative therapeutic agents, in particular, natural derived substances (Liu and McCullough, 2011).

Immediately after ischemia onset, a series of cascading events occur which consequently contribute to the pathological changes and clinical syndromes of stroke. Energy failure due to diminished oxygen and glucose supply cause reduced ATP and ionic imbalance, which subsequently results in membrane depolarization and calcium overload. Glutamate over release further facilitates calcium influx and initiates downstream inflammatory and apoptotic mediators (Barone and Feuerstein, 1999; Wang et al., 2007). The inflammatory cascade occurs within an hour after ischemic stroke and thus occupies a prominent position in the pathophysiology of stroke (Wang et al., 2007; Grønberg et al., 2013). Rapid activation of astrocytes and microglial cells is followed by brain permeation of circulating inflammatory cells, such as granulocytes and leukocytes (Chung and Benveniste, 1990). Moreover, tissue damage due to blockade of blood flow also initiates proinflammatory interleukin-1 (IL-1β), tumor necrosis factor alpha (TNF-α), and interleukin-6 (IL-6), which were released instantly after ischemic onset and are potential mediators of inflammation, compromising clinical prognosis and increasing infarct volume in humans (Vila et al., 2000).

Mitochondrial dysfunction and oxidative phosphorylation decoupling happen early during ischemia and generate numerous reactive oxygen species (ROS), leading to widespread oxidative stress (Dirnagl et al., 1999). ROS like free radicals’ damage lipids, proteins, DNA, and cause intracellular organelle destruction *via* plasma and organelle membrane peroxidation, such as the endoplasmic reticulum and mitochondria. These could further engender the release of biologically active free fatty acids, such as arachidonic acid and DNA fragmentation. Joined by the abovementioned energy failure, glutamate-induced excitotoxicity, and inflammatory factors, the vicious cycles induced by ROS eventually activate the injury pathways and lead to cell necrosis and apoptosis.

Polydatin, also called piceid, is a traditional Chinese medicine that has broad range of pharmacological activities, including the anti-inflammatory and antiapoptotic activity (Gao et al., 2016). The neuroprotective properties of polydatin have been demonstrated in both cerebral ischemia and other neurodegenerative diseases (Cheng et al., 2006; Xu et al., 2016). Previous studies have shown that polydatin successfully counteracts the deleterious effects of ischemic stroke in animal model (Cheng et al., 2006; Ji et al., 2012). Polydatin diminishes infarct volume, reduces brain water, and improves neurologic scores in focal cerebral ischemia (Cheng et al., 2006). Moreover, the neuroprotective effect of polydatin in cerebral ischemia is explored and attributed to several critical molecules involved in inflammation and oxidative stress (Ji et al., 2012), suggesting the biological activities of polydatin cannot be attributed to a single pathway or receptor, but may involve a broad range of pathological processes. Indeed, in renal ischemia, polydatin exerts nephroprotective effects by PI3K/AKT signaling cascade (Liu et al., 2015). In cardiac ischemia, polydatin attenuates oxidative stress by renin–angiotensin system (RAS) and Rho kinase (ROCK) pathways (Ming et al., 2017). In brain ischemia, polydatin acts as neurotropic factor and activates BDNF expression (Sun et al., 2014). Moreover, polydatin downregulates cell adhesion molecules (CAMs) and modulates the migration of inflammatory cell in cerebral ischemic injury (Cheng et al., 2006). Numerous factors in ischemia sequentially mediate the pathological processes, ranging from acute energy failure and glutamate/calcium overload, to subacute neuroinflammation and oxidative stress, eventually causes neuronal cell death. Thus, future neuroprotective agents may warrant multiple steps procedures, as various single target neuroprotective strategies have been tested but yielded disappointing results.

The present study aims to evaluate whether polydatin’s effects on neuroinflammation and oxidative stress could eventually account for cellular protection. If so, the potential molecular and cellular mechanisms underlying these effects still merit further delineation. Results obtained will not only help us to understand the cascading mechanisms eventually leading to cell death, but also provide a clue as to the potentials of multiple targeting therapeutics.

## Materials and Methods

### Animal Grouping and Drug Treatment

Adult male Sprague–Dawley rats weighing 230 to 260 g, 7 to 10 weeks were purchased from Guangdong Medical Laboratory Animal Center, China. The experimental animals were housed at Laboratory Animal Research Center, Peking University Shenzhen Graduate School, under 12 h light/12 h dark cycle at 18°C to 22°C and had free access to diet and tap water throughout the study. The experimental procedures were set in such a way to minimize rats suffering. All experimental procedures were carried out according to the protocols approved by Institutional Animal Care and Use Committee of Peking University Shenzhen Graduate School. The rats were randomly divided into four groups (n = 10–20/group): 1) Vehicle treated control group/Sham; 2) Middle cerebral artery occlusion group/MCAO; 3) Rat undergoing MCAO and treated with polydatin/Poly+MCAO; 4) Sham-operated group and treated with polydatin/Poly+Sham. Two doses of polydatin (30 mg/kg; Sigma) or vehicle were administered intraperitoneally. The first dose was administered 30 min before ischemia, and the second dose was administered within 1 h after ischemia, in accordance with previously reported protocols (Cheng et al., 2006; Ji et al., 2012).

### Middle Cerebral Artery Occlusion Surgery

MCAO was carried out as we have described previously (Shah et al., 2014; Park et al., 2018). The two main branches of right common carotid artery, external and internal carotid arteries, were exposed. The two smaller braches of external carotid artery, occipital artery and superior thyroid artery, were knotted with 6/0 black silk and subsequently pierced. A blue nylon filament (3/0) approximately 30 mm in length had having a blunt, rounded tip was inserted from the external carotid artery into internal carotid artery and advanced further into the origin of the MCA. After 24 h of permanent occlusion, the animals were decapitated, and brain tissues were collected. In sham-operated animals, all these procedure were performed with exception of nylon insertion.

### Neurobehavioral Test

A five-point scale was used to describe neurologic deficit scores. Rats showing no sign of injury received a score of 0, while a score of 1 represented minor neurologic deficits. Moderate and severe neurologic deficits, in which rats showed circling movements (mild to spontaneous circling) with depressed consciousness, were graded from 2 to 4. Rats receiving scores from 2 to 4 were processed for further experimentation. Under this criterion, seven rats were eliminated from further testing: five from group 2 and two from group 3.

### Chloride Staining and Histological Preparation



After being evaluated for neurological deficits, rats were decapitated under anesthesia, and brain tissues were carefully removed. Three-millimeter-thick coronary sections were cut from the frontal lobe with a sharp blade. Slices were incubated in 2% 2,3,5-triphenyltetrazolium chloride (TTC) for 10 to 20 min in a water bath at 37°C until a thorough demarcation was observed, and then fixed in 4% paraformaldehyde solution and photographed (Shah et al., 2018). The infarct area was then measured with ImageJ program and expressed as a percentage to total area.

To compensate for brain edema, the corrected brain infarction was calculated as following:

Corrected infarct area=left hemisphere area−(right hemisphere area−infarct area)

Brain sections were imbedded in paraffin, and 4-μm coronary sections were made by using a rotary microtome. The slides were deparaffinized in xylene and rehydrated in graded alcohol and proceeded to the following staining techniques. 

### Cresyl Violet Staining

Tissue slides were rinsed with distilled water and 0.1 M phosphate buffer saline (PBS), and stained with 0.5% cresyl violet solution, followed by rinsing with distilled water and differentiated in ethyl alcohol (70%, 95%, and 100%). Brain slides were cleared with xylene and mounted with a glass cover slip. The stained images were pictured with light microscope (Olympus, Japan), saved as TIF files, and analyzed by ImageJ computer-based program. The TIF images were optimized to the same threshold intensity for all groups for pyknotic, red, and ghost neurons, while focusing specifically on neuropil, neuronal size, and shape (frontal and piriform cortex).

### TUNEL Staining

Terminal deoxynucleotidyl transferase (TdT) dUTP Nick-End Labeling (TUNEL) assay staining was performed with a commercial kit according to the manufacturer’s protocol (Oncogene Research Products, Cambridge, MA). The deparaffinized sections were permeated with proteinase K, after which the peroxidases were inactivated. The slides were subsequently incubated in equilibration buffer with terminal deoxynucleotidyl transferase (TdT) labeling reaction mixture at 37°C for 1.5 h. This reaction was stopped by stopper buffer. The sections were washed with 0.1 M PBS and stained in 3,3′-diaminobenzidine tetrahydrochloride (DAB) solution, washed with distilled water, dehydrated in graded ethanol (70%, 95%, and 100%), fixed in xylene, and cover-slipped in mounting medium. Immunohistochemical TIF images were captured with a light microscope (five images per slide) and TUNEL-positive cells were quantitated with ImageJ software. TIF images were optimized to the same threshold intensity for all groups and was expressed as the relative TUNEL neuronal cells/section of the samples relative to the control (Shah et al., 2019).

### Immunohistochemical Analysis

Immunohistochemical staining was performed as previously described with minor modification (Sung et al., 2012). The slides were processed for the antigen retrieval step (enzymatic method), then washed with PBS. The endogenous peroxidase was quenched by applying 3% hydrogen peroxide (H_2_O_2_) in methanol for 10 min. The slides were incubated with 5% normal goat serum containing 0.1% Triton X-100. After being blocked, the slides were incubated overnight in mouse anti-phosphorylated-c-Jun N-terminal kinase (p-JNK), rabbit anti-IL-1β, rabbit anti-p-nuclear factor-κB (NF-κB), and rabbit anti-iNOS antibodies (Santa Cruz Biotechnology, Inc). The following morning, after being washed with 0.1-M PBS, the slides were incubated in biotinylated secondary antibodies according to the origin of the primary antibody and serum used, then incubated with ABC reagents (SCBT USA) for 1 h in a humidified chamber. The slides were washed with 0.1-M PBS, stained in DAB solution, washed with distilled water, dehydrated in a graded ethanol series, and cover-slipped in mounting medium. P-JNK, IL1-β, p-NF_K_B, and iNOS in cortex/total area were quantitatively determined as above.

### Immunofluorescence Analysis

Slides were treated with proteinase K (antigen retrieval step), washed with 0.1 M PBS, and incubated with 5% normal serum according to the source of the secondary antibody used. The slides were incubated with primary antibodies against glial fibrillary acidic protein (GFAP), TNF-α, ionized calcium-binding adapter molecule 1(Iba-1), p-NF-κB, thioredoxin (TRX), and 8-oxoguanine (Santa Cruz Biotechnology, Inc), overnight at 4°C. The next morning, after being washed with PBS, the slides were incubated with fluorescently labeled secondary antibodies (Santa Cruz Biotechnology) for signal amplification in a dark chamber, then cover slipped in Ultra Cruz mounting medium (Santa Cruz Biotechnology, Inc). Immunofluorescence images (five images per slide) were captured using confocal scanning microscopes (Flouview FV 1000; Olympus, Japan), and the same region of the cortex/total area for all groups were quantitated as above.

### Western Blot Analysis

This procedure was performed as previously described (Shah et al., 2015), the samples were dissolved in lysis buffer (1 M Tris HCl, 5 M sodium chloride, 0.5% sodium deoxycholate, 10% sodium dodecyl sulfate, 1% sodium azide, 10% NP-40), and phenylmethylsulfonyl fluoride (PMSF) was added as a protease inhibitor. The homogenate was centrifuged, and the protein concentration was determined with a bicinchoninic acid (BCA) kit (Pierce, Rockford, IL) according to the guidelines provided by the manufacturer. Equals amount of protein (i.e., 30 µg per sample) were electrophoresed on 10% sodium dodecyl sulfate polyacrylamide gel electrophoresis (SDS-PAGE) gels followed by immunoblotting to transfer the protein to polyvinylidene fluoride (PVDF) membranes (Millipore, Billerica, MA). To minimize nonspecific antibody binding, we blocked the PVDF membranes with skim milk for 1 h at room temperature. The PVDF membranes were washed in Tris-buffered saline containing 0.1% TWEEN 20 (TBST) and then incubated with primary antibodies overnight at 4°C. The membranes were then incubated with appropriate secondary antibodies, and the protein bands were detected using an ECL detection reagent according to the manufacturer’s instructions (Amersham Pharmacia Biotech, Piscataway, NJ). The antibodies used include anti-p-JNK, JNK, anti-TNF-α, anti-IL-1β, anti-Toll-like receptor 4 (TLR4), anti-cycloxygenase-2 (COX2), anti-iNOS, anti-p-NF-κB, anti-nuclear factor erythroid 2-related factor 2 (Nrf2), anti- heme oxygenase-1 (HO-1), anti-TRX, and anti-β-actin (Santa Cruz, Biotechnology, CA, USA), as well as anti-p-P38, P38 (Cell Signaling Technology, CST).

### Statistical Analysis

Western blot bands and immunohistochemical data were analyzed using ImageJ software (ImageJ 1.30; https://imagej.nih.gov/ij/). The data are presented as the means ± SEM, and neurological score data are presented as the median and range. Data were analyzed by one-way analysis of variance (ANOVA) followed by a *post hoc* Bonferroni multiple comparison test using GraphPad Prism 5 software. In all analyses, differences were considered significant at *p* < 0.05. The symbol ∗ indicates a significant difference relative to sham, whereas shows a significant difference relative to MCAO.

## Results

### Effect of Polydatin on Neurological Scores, Brain Infarction, and Neuronal Cell Loss

**Figure 1:**
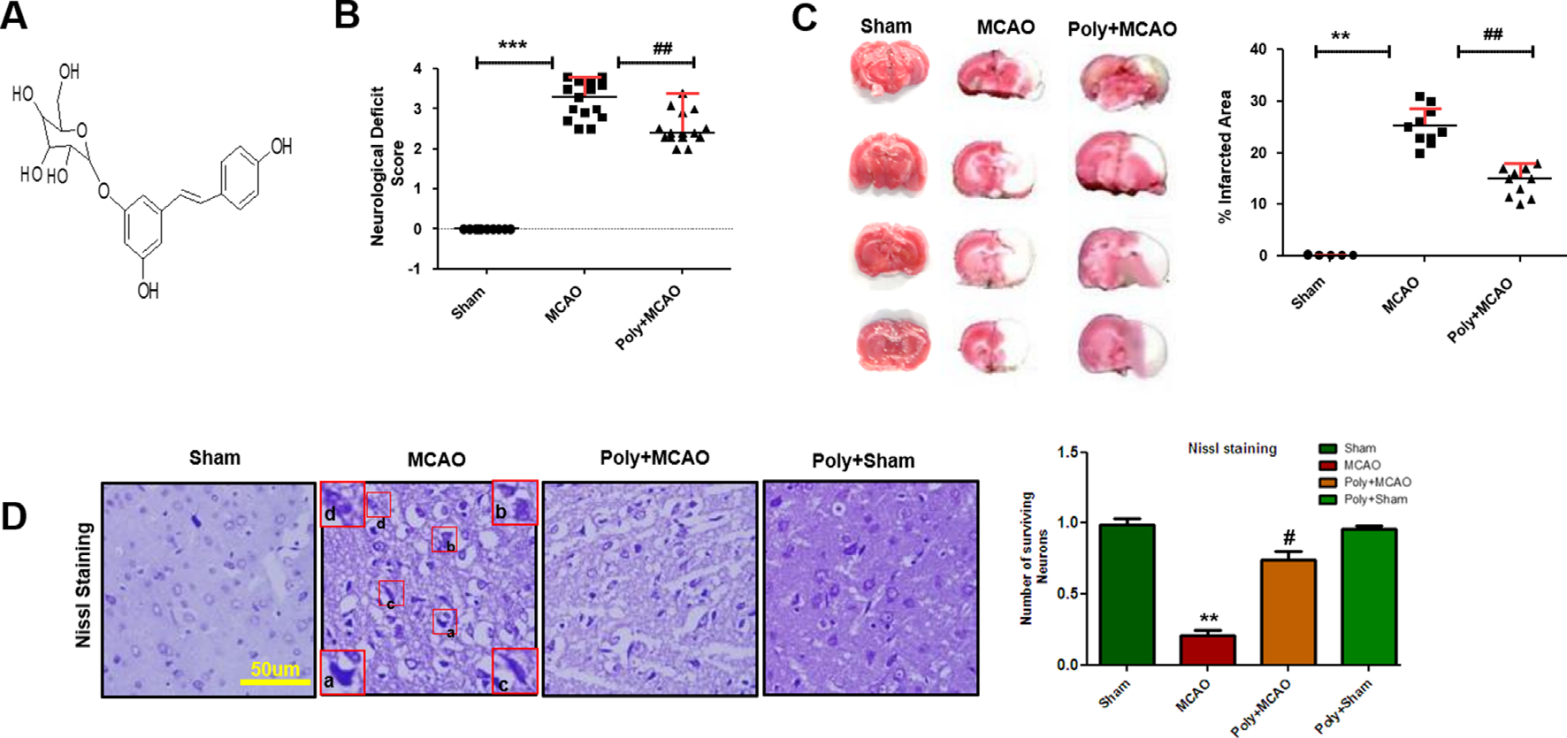
Effect of polydatin on neurological scores, brain infarction and neurodegeneration. **(A)** Structure of polydatin. **(B)** Polydatin attenuated neurological deficits. Neurological test was conducted 24 h after ischemia. Poly+MCAO rats had significantly improvement of neurological deficits (^##^ *p* < 0.01) compared to MCAO group. **(C)** Brain coronal sections were stained with TTC, which distinguishes between ischemic and non-ischemic areas (n = 5–10/group). **(D)** Representative photomicrograph of cresyl violet staining showing the extent of surviving neurons in the cortex, scale bar = 50 μm. (a, b) Cytoplasmic swelling and scalloped neurons with intense cytoplasmic eosinophilia and nuclear basophilia. These changes result from neuronal ischemia, which impairs metabolism in the tissue. (c) Cytoplasmic fading of neurons, which invariably occurs in neurons at later stages of necrosis (ghost neurons). (d) Some cells had shrunken appearance along with pyknotic nuclei. Intensive neuropil vacuolation can be seen in ischemic cortex (MCAO). ***p < 0.001; **, ^##^ *p* < 0.01; ^#^ *p* < 0.05.

Neurological scores were determined 24 h after permanent MCAO. Polydatin structure is illustrated in **Figure 1A**. Animals subjected to MCAO exhibited severe neurological deficits ranging from spontaneous circling (scores, 3 and 4) to depressed consciousness (Shah et al., 2016). Treatment with polydatin attenuated these scores, demonstrating that polydatin can mitigate neurological deficits (*p* < 0.01, **Figure 1B**). The neocortex, caudate, and putamen were the most frequently damaged regions in this kind of ischemic model (Shah et al., 2018). TTC staining entirely demarcated the infarcted area from the deep red area of intact tissue (**Figure 1C**), with sham-operated rats showed no infarction. Polydatin treatment reduced the size of infarction induced by MCAO (*p* < 0.01, **Figure 1C**).

To further examine, Nissl staining was used to reveal the extent of neuronal injury in cortex. A substantial difference was observed in the neuropil of ischemic cortex as compared with sham-operated control, where ischemia caused robust neuronal cell loss in the cortex and polydatin treatment attenuated this damage (**Figure 1D**, *p* < 0.05). Moreover, aberrant morphological neuronal features were identified in the MCAO cortex, including alteration in neuronal size and shape (swelling and a scalloped, angular nature), alteration in color staining (cytoplasmic eosinophilia/pyknosis, nuclear basophilia), and vacuolation (**Figure 1D**).

### Effect of Polydatin on Neuronal Apoptosis and Activation of the p38/JNK Pathways

**Figure 2:**
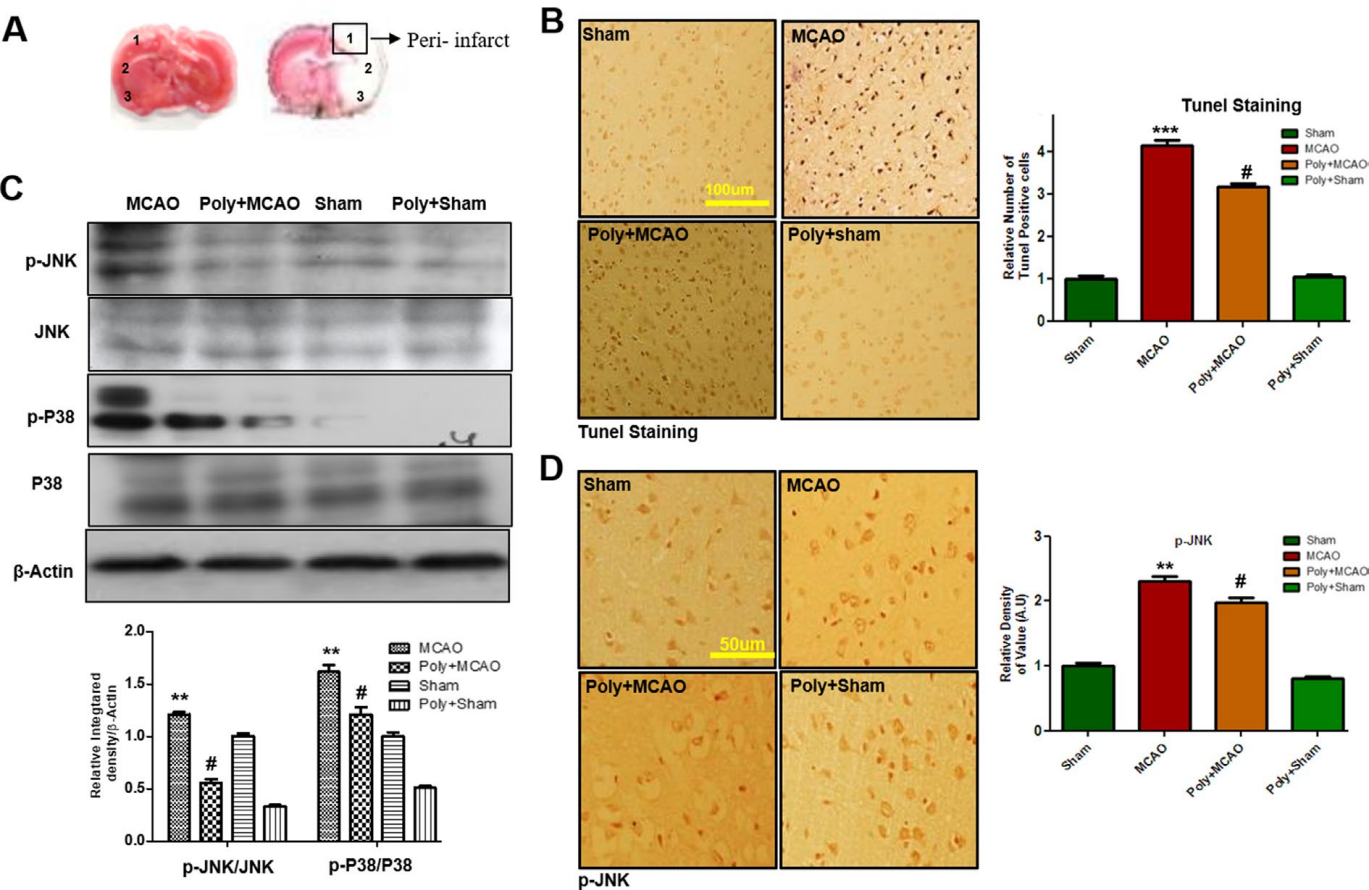
Effect of polydatin on neuronal apoptosis and MAP kinase pathways. **(A)** TTC staining of coronal sections 24 h after permanent MCAO, separated by frontal cortex (1), parietal cortex and insular cortex (2), and periform cortex (3). The analyzed peri-infarct region, region of interest indicated by square 1 **(B)** Representative images of TUNEL histochemistry show apoptotic cells; scale bar = 100 μm. MCAO caused significant neuronal apoptosis, while polydatin treatment attenuated apoptotic damage. Data presented are relative to Sham (n = 3–5/group). **(C)** Polydatin prevents MCAO-induced activation of p-p38 and p-JNK. Representative Western blots of p-p38 and p-JNK (n = 5–8/group) and β-actin was used as a control. **(D)** Immunoreactivity of p-JNK; scale bar = 50 μm. p-JNK exhibits cytoplasmic localization, and number of experiment performed = 3. ***p < 0.001; **p < 0.01; ^#^ *p* < 0.05.

Neuronal cell loss in ischemic injury may result either from necrosis mainly in the core region, where blood flow is reduced by 90% and ATP supply is severely depleted, or from induced apoptosis mostly within the penumbra area. Depending on neuronal cell type, condition, age, and brain location, necrotic and apoptotic cell death pathways have broad crosstalk due to the common triggers, including the calpains, nitric oxide, and poly ADP ribose polymerase (PARP), which are not easily discernable based on morphological changes. We then analyzed peri-infarct cortical region as indicated by square 1 (**Figure 2A**). TUNEL staining was performed to examine the apoptotic cells and the relative effect of polydatin. Considerably, more TUNEL-positive cells were noticed in the peri-infarcted area of the MCAO ipsilateral cortex (*p* < 0.001, **Figure 2B**) than in that of poly+MCAO (*p* < 0.05, **Figure 2B**).

p38 MAPK and JNKs are important members of the MAPK family involved in apoptotic pathways (Kyriakis and Avruch, 2012). Both JNK (as p-JNK) and p38 (as p-p38) activate apoptotic cell death by transcriptional and posttranscriptional modification (Xia et al., 1995). JNKs is critical in death receptor-initiated extrinsic and mitochondrial elicited intrinsic apoptotic pathways, whereas p38MAPK inhibition could protect against apoptosis and necrosis in heart ischemia. To reveal the possible involvement of p38 MAPK/JNK in polydatin’s protective effects against apoptosis, Western blot analysis was performed to investigate the activation of p38/JNK in cortical tissue after occlusion. The results showed elevated expression of activated p38 and JNK in MCAO group as compared with sham-operated controls, which could be reduced by polydatin administration (**Figure 2C**). These results were further validated by immunohistochemical experiments as shown for p-JNK (**Figure 2D**).

### Polydatin Downregulates Ischemia-Related Neuroinflammation

p38 is responsive to stress stimuli, such as inflammatory cytokines and ROS. It is also involved in mediating cytokine production including tumor necrosis factor-α (TNF-α), interleukin-1ß (IL-1ß) in immune cells and regulating pro-inflammatory signaling networks. JNK is involved in both adaptive and innate immune response because it could be activated by various proinflammatory cytokines, such as TNF and IL-1, and Toll-like receptor ligands. Moreover, a feedback mechanism exists between inflammatory cytokines and activated p38/JNK, in which activation of one pathway will activate the other, suggesting critical roles of p38/JNK in inflammation (Nakajima et al., 2004; Tu et al., 2011).

**Figure 3:**
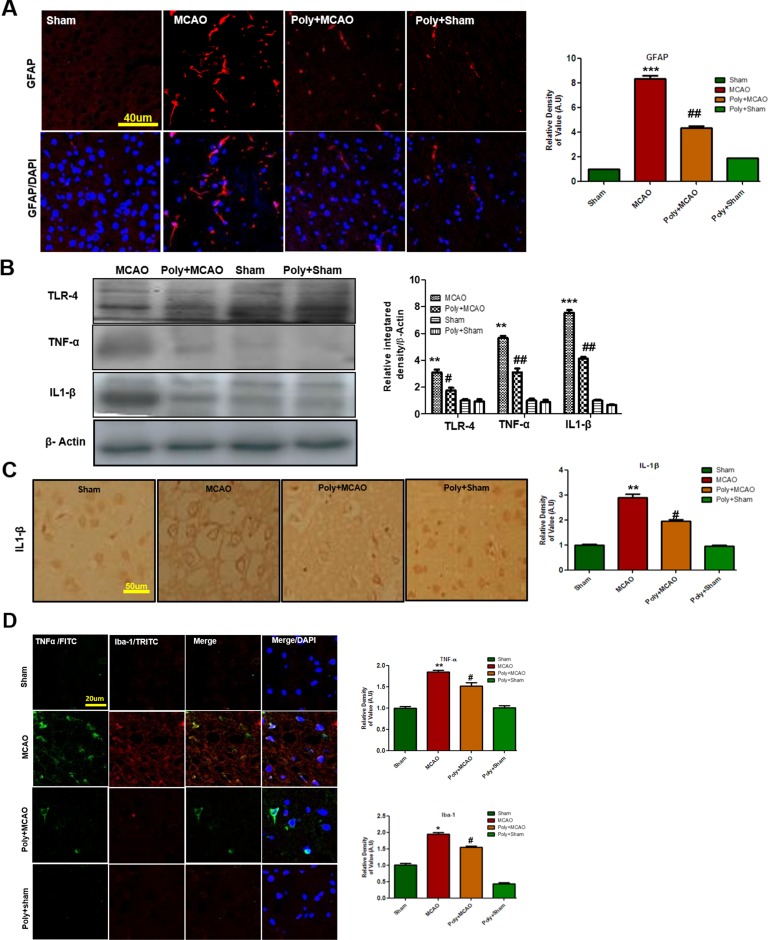
Polydatin attenuated reactive gliosis and inflammatory mediators in MCAO injury. **(A)** Immunoreactivity of astrocytes (GFAP-positive cells) in the sham, MCAO, and Poly+MCAO groups are shown. Scale bar = 40 μm. The GFAP-positive cells were visualized by TRITC. Data presented are relative to Sham and number of experiment performed = 3, and (n = 5–7/group). **(B)** Effect of polydatin on inflammatory cytokines. Western blot analysis of TLR4, TNFα, and IL-1β (n = 5–8/group). The Western blots were quantified using ImageJ and analyzed by GraphPad Prism 5 software. β-Actin was used as a control. **(C)** Immunohistochemistry results for IL-1β are shown; scale bar = 50 µm. **(D)** Double immunofluorescence reactivity of TNF and Iba-1. Scale bar = 20 μm. The cortical sections show correspondingly elevated expression of TNF and Iba-1 after 24 h of permanent ischemia. TNF is visualized with FITC and IBa-1 by TRITC (n = 5-7/group). ***p < 0.001; **, ^##^ *p* < 0.01; ^#^ *p* < 0.05.

Ischemic stroke is characterized by reactive gliosis in which the residing microglia and astrocytes assume characteristic cellular appearances to mediate progression of ischemic injury. Following this, we investigated the involvement of astrocytic (GFAP-reactive cells) and microglial activation (Iba-1-reactive cells) in cortex. Immunofluorescence results revealed significant increases in the numbers of GFAP- and Iba-1-reactive cells in MCAO cortex compared with sham cortex, an effect that could be downregulated by polydatin treatment (**Figure 3A**).

Both of these activated hypertrophic cells work as resident machinery for generating inflammatory mediators and cytokines that are implicated in ischemia (Chung and Benveniste, 1990; Yu and Lau, 2000). Reports have consistently supported the involvement of TLR4 in inflammation in ischemic brain injury (Bowman et al., 2003; Caso et al., 2008). Western blot analysis was performed to evaluate the expression of TLR4 24 h after permanent MCAO. Our results showed that ischemic brain injury significantly elevated the expression of TLR4, which could be effectively decreased by polydatin (**Figure 3B**). Next, we examined the expression levels of TNF-α and IL-1β, which increased within hours after ischemia (Davies et al., 1999). Our results revealed that ischemic brain injury significantly elevated their expression in ischemic cortex compared with the sham-operated controls (**Figure 3B**). The increasing effects of ischemic stroke could be reversed by polydatin (**Figure 3B**). Likewise, immunohistochemistry of IL-1β (**Figure 3C**) and double immunofluorescent colocalization of TNF-α in Iba-1 immunopositive cells demonstrated higher expression of both in the cortex of MCAO group (**Figure 3D**). Polydatin treatment significantly decreased the expression of these inflammatory markers (**Figure 3C, D**).

### Effect of Polydatin on Oxidative Stress

**Figure 4:**
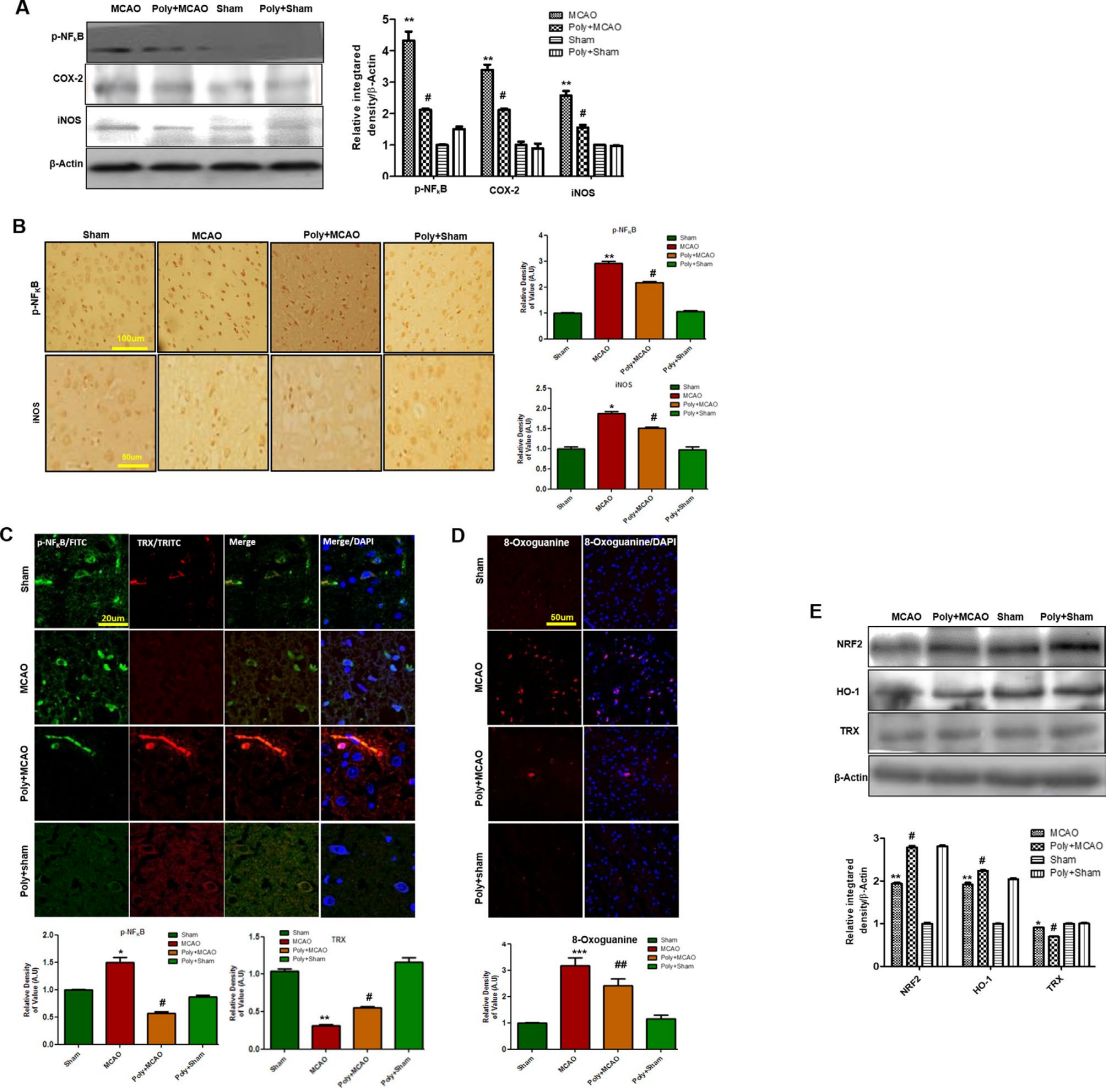
Effect of polydatin on oxidative stress. **(A)** Western blot analysis of COX2, iNOS, and p-NF-κB (n = 5–8/group). The protein bands were quantified using ImageJ and analyzed with GraphPad Prism 5 software. β-Actin was used as a control. Densitometric analysis was expressed in arbitrary units. **(B)** Representative photos of immunohistochemistry for p-NF-κB and iNOS; scale bar = 50 µm. **(C)** Double immunofluorescence reactivity of p-NF-κB and TRX. Scale bar = 20 μm and (n = 5–7/group). The ischemic tissue showed elevated expression of p-NF-κB and attenuated expression of TRX after 24 h of continuous ischemia. p-NF-κB and TRX were visualized by FITC and TRITC, respectively. **(D)** Representative immunofluorescence images of 8-oxoguanine staining (n = 5–7/group). Scale bar = 50 μm. **(E)** Polydatin treatment increased the levels of several antioxidant proteins in ischemic cortex. Western blot analysis of Nrf2, HO-1, and TRX (n = 5–8/group). The protein bands were quantified using ImageJ and analyzed by GraphPad Prism 5 software. β-Actin was used as a control. ***p < 0.001; ^##^, **p < 0.01; ^#^, *p < 0.05.

Stress signals, such as proinflammatory factors TNFα, IL-1β, or Toll, bind to their corresponding receptors that further link to downstream molecules, involving the sequential activation of ASK1, SEK1, and JNK. This process is followed by the proteasome-mediated degradation of IκBs, activation, and nuclear translocation of NF-κB, where it induces transcription of ROS generation-related molecules, such as iNOS and COX2 (Yao et al., 2013). Accordingly, Western blotting results showed elevated expression of these mediators in ischemic brain (*p* < 0.01, **Figure 4A**), and polydatin treatment significantly mitigated their expression (*p* < 0.05). These results were further validated by immunostaining findings for p-NF-κB and iNOS (**Figure 4B**). Activation of p-NF-κB encodes the bulk of inflammatory mediators that exacerbate ischemic brain injury. To demonstrate the association between inflammatory p-NF-κB and thioredoxin (TRX), a small thiol-active protein important for maintaining intracellular redox status in the ischemic brain, we performed double immunofluorescence (**Figure 4C**). Our results showed elevated expression of p-NF-κB in ischemic cortex and correspondingly lower expression of TRX. Treatment with polydatin significantly reduced the expression of p-NF-κB while increasing TRX expression. Moreover, 8-oxoguanine as an oxidative stress marker plays a vital role in the pathogenesis of ischemic brain injury (Ali et al., 2015). We then measured oxidative stress in ischemic brain by using 8-oxoguanine fluorescence staining. Our results demonstrated elevated expression of 8-oxoguanine in ischemic cortex, and this increase would be attenuated by polydatin treatment (**Figure 4D**).

Nrf2 is a ubiquitously expressed transcription factor that serves vital protective functions *via* regulating the expression of various detoxifying and antioxidant process-related proteins. Activated Nrf2 translocates to the nucleus and stimulates transcription of several downstream antioxidant proteins, such as HO-1, superoxide dismutase (SOD), and glutathione (GSH). The migration pattern of Nrf2 on SDS-PAGE electrophoresis is intensively debated. Recently, it has been found that the Nrf2 band may be located at approximately 90 to 110 kDa (Lau et al., 2013). Our Western blot results demonstrated elevated expression of Nrf2 and HO-1 in ipsilateral cortex after 24 h of permanent MCAO relative to the sham controls, and polydatin significantly attenuated this change (**Figure 4E**). Noticeably, we found a high-intensity band for Nrf2 (90–120 kDa) and HO-1 in the polydatin-treated group, with the same bands appearing at low intensity in the sham group (**Figure 4E**).

## Discussion

The present study demonstrated that polydatin treatment attenuated neuronal cell loss in ischemia *via* rescuing apoptotic cell death in MCAO, which are likely mediated by ameliorated neuroinflammation, reduced oxidative stress, and enhanced antioxidant mechanisms. Accumulation of toxic radicals, such as ROS triggered oxidative stress, is closely related to neuroinflammatory activation, which further exacerbates ischemic pathogenesis. Polydatin treatment had strong protective effects not only through diminishing pro-neuroinflammatory factors and p-NF-κB–mediated ROS generation, but also strengthened intrinsic antioxidative mechanism, including Nrf2, HO-1, and TRX. In clinical scenarios, thrombolytics, such as activase and TNKase, remained the few available pharmacological choices for clinical application, and the usefulness of which is severely restricted within 3 to 4.5 h of the onset of ischemia due to its short therapeutic window, and thus, a limited patient can be benefited. Combination of low-dose aspirin and other anti-coagulants is an established prophylactic regime for stroke. However, the use of these thrombolytics abolish only the occlusion but lack the efficacy to attenuate tissue damage induced by ischemic injury. Therefore, neuroprotective agents are required to stabilize the physiological function of brain. As repetitive clinical trials of neuroprotectants targeting a single step of stroke pathological process have failed previously, polydatin in this study ameliorated the detrimental effects of MCAO by targeting multiple stages of stroke, such as neuroinflammation, oxidative stress, and neuronal apoptosis. Our results demonstrated that a neuroprotectant acting at different stages may be more advantageous to intervene in the vicious cycles in MCAO.

Inflammation worsens the clinical prognosis of stroke and compromises its therapeutic outcome. Stroke triggers both resident and peripheral cells to migrate to the brain parenchyma and contribute to inflammatory pathogenesis (Grønberg et al., 2013). Release of inflammatory mediators substantiates ischemia-induced neuronal death by several mechanisms. First, activation of TLR-4 on glial cells stimulates stress-related kinases, such as JNK and p38-MAPK (Kacimi et al., 2011), whose activation triggers the mitochondrial apoptotic pathway. Second, following these kinases activation, inflammatory cytokines are produced largely by activated microglia, astrocytes, and neurons within the first hour after ischemic injury (Rothwell, 1991; Putcha et al., 2003; Munoz et al., 2007; Wang et al., 2012). Third, activated nuclear transcriptional machinery like NF-κB ultimately elicits the production of pro-inflammatory factors, such as TNFα, IL-1β, and nitric oxide, further aggregating the damage by the initial pathological stimuli. The higher expression of NF-κB and TLR-4 in our study was in agreement with previous reports that TLR-4 receptors have cytotoxic roles in ischemic brain injury, and TLR-4 knockdown is linked to reduced infarction area (Caso et al., 2007).

It has been reported that NF-κB activation by TLR-4 triggers iNOS, COX-2 production (Yao et al., 2013). Both COX-2 and iNOS are toxic mediators of inflammatory cascade whose activity can be down-regulated by inhibiting TLR-4 (Caso et al., 2007). In accordance to results from other models, polydatin attenuates ischemia-induced expression of TLR4, NF-κB, COX-2, iNOS, indicating its anti-inflammatory effects (Huang et al., 2017; Tang et al., 2018). In our study, ROS activity in ischemic cortex were assessed using 8-oxoguanine as marker. Increased 8-oxoguanine expression in ischemic tissue was significantly attenuated by polydatin, showing the antioxidant nature of polydatin in our ischemic model. The detailed molecular mechanisms undying polydatin’s effects are yet unknown but could be partially attributed to its free radical scavenging properties. Recently published data demonstrated that polydatin administration is highly useful to cope against various models of (both *in vivo* and *in vitro*) toxicities, or against neuronal death in numerous kinds of ischemic models (Cheng et al., 2006; Zhang et al., 2012; Ji et al., 2012).

Interestingly, a strong correlation between inflammation and oxidative stress is demonstrated in both neuronal and nonneuronal models (Yeligar et al., 2010; Ali et al., 2018). Studies reiterating the free radical scavenging and antioxidant profile of polydatin show that polydatin treatment restores several antioxidant enzymes in different disease models (Qiao et al., 2016). Thus, the ubiquitous anti-oxidative approach of polydatin can be largely attributed to its mobilizing endogenous antioxidant systems, such as activation of SODs, GSH, sirtuins (Sirt), Nrf2/HO-1. Indeed, previous studies demonstrate that polydatin is a potent activator of Sirt1, Sirt3, Sirt6, which are involved in various cellular processes including cell survival by promoting SOD and other antioxidant proteins (Caso et al., 2007). One recent study suggested polydatin induces SIRT1-Nrf2 pathway, as is also true for other polyphenol substances (Huang et al., 2015; da Cunha and Arruda, 2017). TRX is an antioxidant protein that participates in the eradication of ROS, reducing apoptosis and excitotoxicity in ischemic brain injury. As a stress sensor, cytoplasmic TRX has a strong inhibitory effect on NF-κB nuclear translocation (Schenk et al., 1994; Hirota et al., 1999). Our double immunofluorescence results, as were also elaborated in the Western blot results showed that ischemic injury attenuated the expression of TRX but increased the nuclear translation of activated NF-κB. Polydatin treatment reserved TRX activity and downregulates NF-κB. Nrf2 is a physiological antioxidant sensor that dimerizes with Keap1. Upon activation, Nrf2 is set free to translocate to the nucleus and induce other antioxidant genes, such as HO-1 and SOD (Jung and Kwak, 2010). Increased Nrf2 and HO-1 activity begin immediately after ischemia and continue for 24 h (Yang et al., 2009). Polydatin increased the expression of these antioxidant proteins in ischemic cortex, which was also supported by a recent finding that overexpression of Nrf2 and HO-1 bolsters the endogenous defense machinery that competes against oxidative stress in ischemic models (Yang et al., 2009).

In conclusion, MCAO injury activates several proinflammatory mediators including NF-κB and is further linked to ROS generation. Polydatin attenuated MCAO-induced oxidative stress and inflammatory cascade, possibly by modulating ROS/Nrf2/TRX and TLR4/NF-κB pathway, eventually accounting for its neuroprotective effects against neuronal apoptosis.

## Ethics Statement

All experimental procedures were carried out according to the protocols approved by Institutional Animal Care and Use Committee of Peking University Shenzhen Graduate School.

## Author Contributions

FA and TL managed the experimental work. FA, FL, and TL performed surgery, western blot, morphological experiments; performed data analysis. AK, IH, and QZ provide technical assistance. AZ, KPO, LA, FL, QZ, TL, YJ, and SL supported the study, designed study, and wrote the manuscript. YJ and SL are the corresponding authors, reviewed and approved the manuscript and held all the responsibilities related to this manuscript. All authors reviewed and approved the manuscript.

## Funding

This work was supported by grants JCYJ20150529153646078, JCYJ20170412150845848, and JCYJ20170810163329510 by Science and Technology Innovation Committee of Shenzhen; Shenzhen Peacock Innovation Team Grant KQTD2015032709315529; Shandong Provincial Natural Science Foundation of China No: ZR2017MH027; and Shaanxi Key Project on Science and Technology (2017SF-040).

## Conflict of Interests Statement

The authors declare that the research was conducted in the absence of any commercial or financial relationships that could be construed as a potential conflict of interest.
